# Management of anterior thigh injuries in soccer players: practical guide

**DOI:** 10.1186/s13102-022-00428-y

**Published:** 2022-03-18

**Authors:** Lasse Lempainen, Sandra Mechó, Xavier Valle, Stefano Mazzoni, Jose Villalon, Marco Freschi, Luca Stefanini, Alvaro García-Romero-Pérez, Maria Burova, Pavel Pleshkov, Ricard Pruna, Giulio Pasta, Jussi Kosola

**Affiliations:** 1 Sports Trauma Research Unit, FinnOrthopaedics, Joukahaisenkatu 6, 20520 Turku, Finland; 2 Department of Physical Activity and Health, Paavo Nurmi Centre, University of Turku, Turku, Finland; 3 Radiology Department, SCIAS-Hospital de Barcelona, Barcelona, Spain; 4 FC Barcelona, Medical Services, FIFA Center of Excellence, Barcelona, Spain; 5 Football Club AC Milan, Milan, Italy; 6 Football Club Atlético Madrid, Madrid, Spain; 7 Football Club FC Juventus, Turin, Italy; 8 Injury Prevention and Rehabilitation Department, Watford FC, Watford, England; 9 Physiotherapy Department, Universidad Camilo José Cela, Madrid, Spain; 10 Football Club FC Zenit, St. Petersburg, Russia; 11 Medical Department, Parma Calcio 1913, Parma, Italy; 12 Department of Surgery, Kanta-Häme Central Hospital, Hämeenlinna, Finland

**Keywords:** Thigh injury, Soccer, Rectus femoris, Quadriceps, Rupture, Rehabilitation, Surgery

## Abstract

Most of the anterior thigh injuries are contusions or strains, however, some of these injuries can be career ending. Early diagnosis and correct treatment are key to successful outcome. Analyzing injury mechanism and adding both clinical and imaging findings, clinicians can make the right treatment decisions already often in the acute phase of the injury. Low grade contusions and muscle strains are treated well with planned rehabilitation, but complete tendon injuries or avulsions can require operative treatment. Also, neglected minor injuries could lead to chronic disabilities and time lost from play. Typical clinical presentation of anterior thigh injury is swelling and pain during hip flexion or knee extension. In more severe cases a clear gap can be palpated. Imaging methods used are ultrasound and magnetic resonance imaging (MRI) which are helpful for clinicians to determine more exact the extent of injury. MRI can identify possible tendon retractions which may need surgery. Clinicians should also be aware of other traumatic lesions affecting anterior thigh area such as myositis ossificans formation. Optimal treatment should be coordinated including acute phase treatment with rest, ice, and compression together with designed return-to-play protocol. The anatomical structure involved lines the treatment pathway. This narrative review describes these more common reasons for outpatient clinical visits for anterior thigh pain and injuries among soccer players.

## Introduction

Anterior thigh injuries vary from simple strains to career ending tendon ruptures. By acknowledging the injury mechanism, anatomy, treatment options and differential diagnosis, the physician can make correct decisions to optimize athlete’s return-to-play.

Anterior thigh injuries can often be treated conservatively and closely followed by rehabilitation. However, not all anterior thigh injuries are typical and fast-healing muscle injuries [1]. If the wrong treatment method is chosen, the return to play may be considerably delayed. In the worst-case scenario, a seemingly harmless injury may result in a spiral of injuries and eventually to operative treatment, even though systematic and well-conducted rehabilitation could have healed the injury.

The treatment method, prognosis, and time required for rehabilitation may vary considerably depending on the location of the injury and the anatomic structure involved [1]. As in other muscle groups, when the athlete describes a combination of pain and audible noise or sometimes the feeling that something just displaced in the hip during the trauma, a tendon structure injury should be suspected [2, 3]. Often, this type of injury has a different prognosis and even different therapeutic approaches [3–5]. The key to successful and optimal treatment is the correct and early diagnosis.

This practical management study was gathered from relevant literature and deals with the most common anterior thigh injuries, their clinical findings, diagnosis, therapeutic options, and complications.

## Practical management of anterior thigh injuries

### Epidemiology, etiology and predisposing factors

Quadriceps muscle injuries are the third most common muscle injury group among soccer players, following hamstring and adductor injuries. However, quadriceps injuries can lead to a longer absence of activity compared to hamstring injuries [6]. Most of the indirect injuries to the quadriceps affect only the rectus femoris muscle [1].

Quadriceps muscle injuries account for 5% of all injuries sustained by soccer players and 19% of all muscle tendon injuries [6]. The majority of these injuries are located in the upper or lower third of the rectus femoris muscle.1 Rectus femoris injuries are usually associated with forceful kicks, but they also occur in fast sprints and jumps [7]. These movements also often involve a forceful eccentric contraction of the rectus femoris, in which the muscle is stretched to the maximum.

**Fig. 1:**
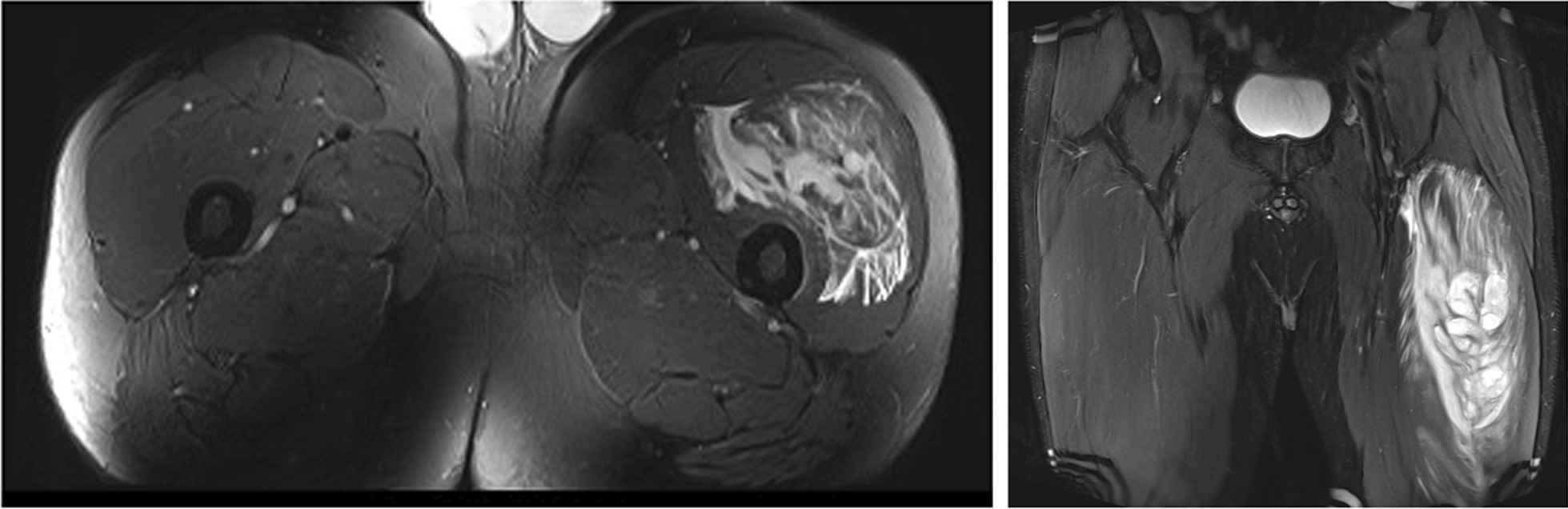
Soccer player with a charley horse of the left thigh due to a quadriceps contusion. Coronal proton density fat saturated MRI image that shows desestructured rectus femoris and vastus lateralis, and a huge intra and intermuscular hematoma

A considerable portion of muscle injuries of the anterior thigh consist of direct contusions, which may be sustained during tackling or other collisions. These injuries are also called a “Charley horse” or a “dead leg” in the literature (Fig. 1). In direct injuries, pain is located at the place of the trauma, and the severity is determined by the amount of energy, surface of the impact, and the contracted/relaxed state of the muscles [7]. In direct contusions, the most frequently affected muscles are the vastus intermedius, vastus lateralis, and vastus medialis [2].

Traditionally, the severity of a "dead leg" can be assessed based on the extent of limping and the deficiency of knee flexion, which correlates with the extent of muscle tissue trauma [8, 9]. The contusions can be classified as mild, moderate, or severe depending on functional strength deficit and evaluating the passive range of motion (ROM) of the knee (> 50% of the knee flexion, < 50% ROM > 30%, or < 30%). The athlete should be re-assessed 24 h post-injury because after contusion, the pain is high and can lead to severe functional deficits. The prognosis of a direct trauma can vary from to 2–5 days in mild cases to 20–25 days or more in severe cases [10].

A previous injury constitutes the greatest risk factor for rectus femoris rupture, in the same manner as in hamstring injuries [11]. Rectus femoris has a high proportion of fast (type II) muscle fibers, during running and kicking it is exposed to significant and often extreme stresses [12, 13] of stretching, powerful eccentric contraction, and power concentric contraction. The risk of recurrent rectus femoris injury is approximately 17% [7]. Poor mobility (reduced hip extension and flexion as well as knee extension) and a previous hamstring injury have been reported to increase the risk of rectus femoris injury [14]. Leg dominance could be one of the risk factors in soccer as quadriceps strains seems to be more common in dominant kicking leg [6, 11]. Surface of game played might be considered as a risk factor thus Woods et al. [15] showed more injuries related to dry fields. Other factors such as body composition, lower extremity strength or age are still debated [7].

### Clinical overview

When examining a quadriceps injury, it is important to know the mechanism of the injury and estimate the location of the injury. The mechanism of the injury indicates whether the injury is a direct contusion of muscle/tendon rupture that occurs during shooting or sprinting. Different muscle injury classifications have been created—Munich, ISMuLT and British muscle injury classifications which summarizes the mechanism of injury, locations, severity and re-injuries [16–18].

**Fig. 2:**
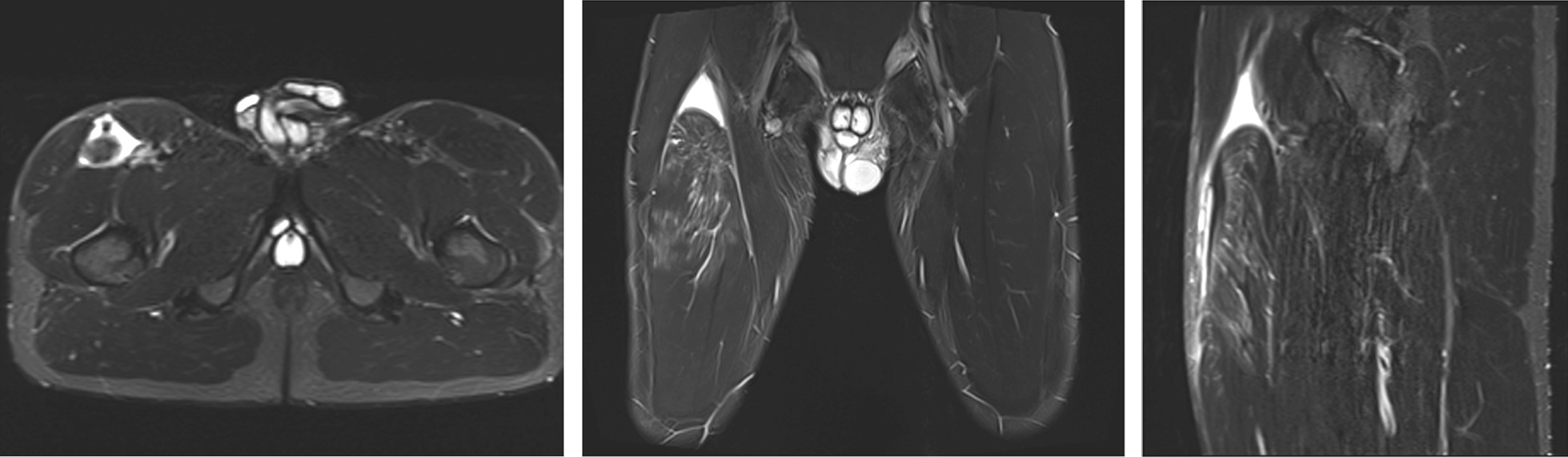
Coronal proton density fat saturated MRI image that shows a complete right proximal rectus femoris rupture at the conjoined tendon level with severe distal retraction of the muscle

**Fig. 3:**
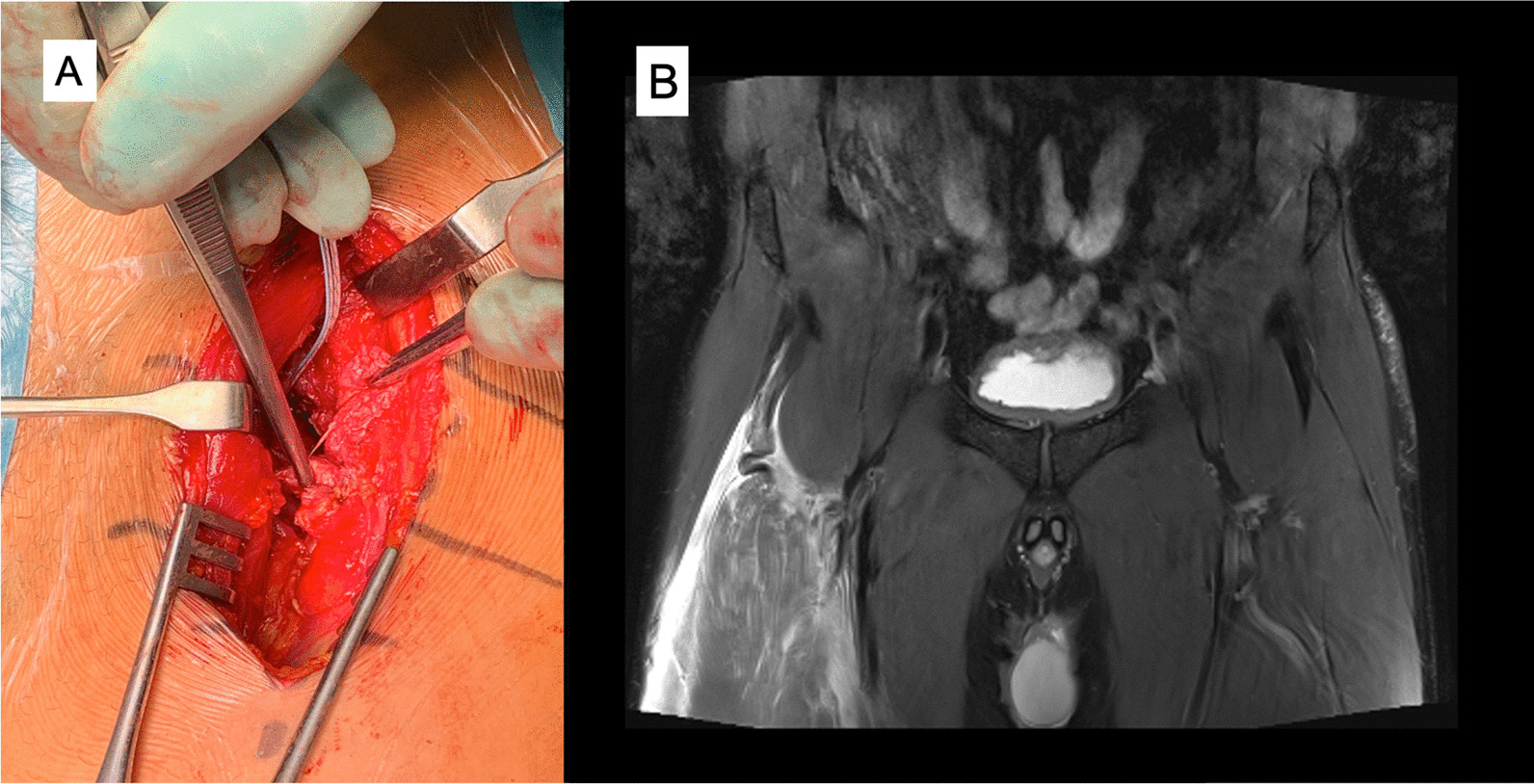
Complete proximal rectus femoris tendon rupture: operative imaging (**A**) and coronal proton density fat saturated image that shows distal retraction of the muscle and the “waving” pattern of the tendon lost tension MRI (**B**)

**Fig. 4:**
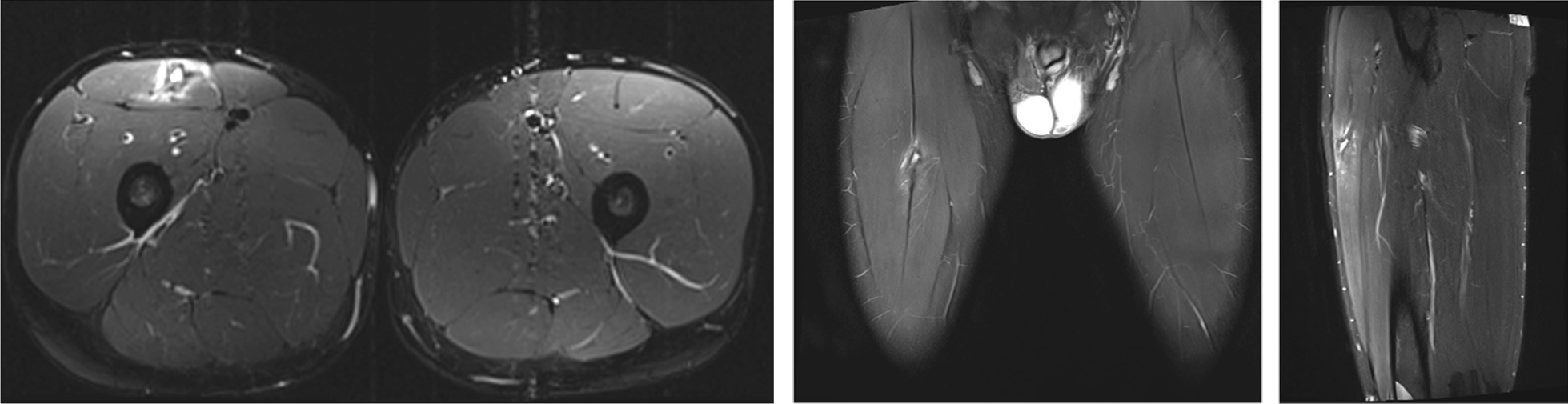
Coronal proton density fat saturated image that shows a persistent hematoma and a rupture in the central tendon. Distal to the hematoma the connective tissue shows scar changes and a mild loss of tendinous tension (MRI)

**Fig. 5:**
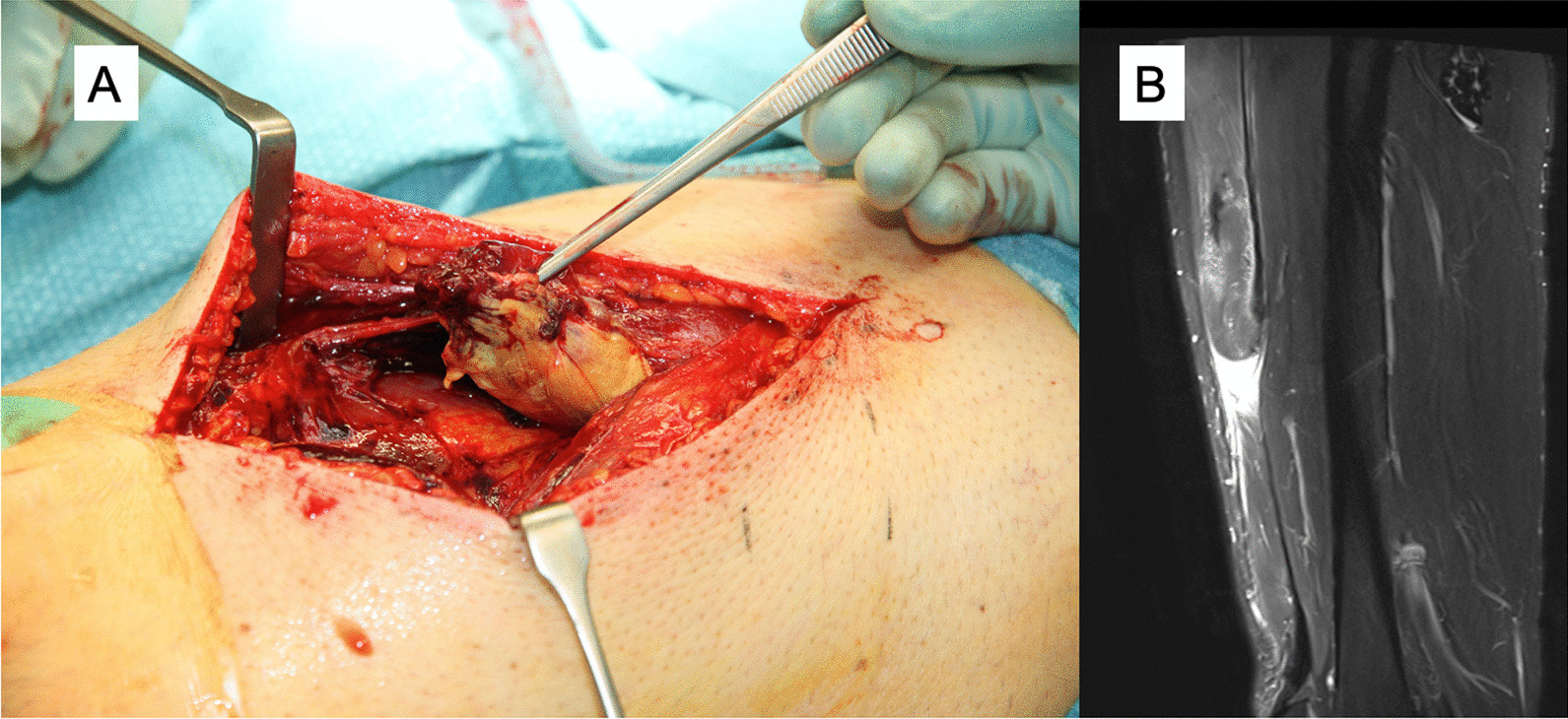
Complete rectus femoris midsubstance rupture: operative image (**A**) and sagittal proton density fat saturated image that shows a complete distal myotendinous junction rupture and an anterior myofascial rupture. Moderate proximal retraction of the muscle. Proximal to the rupture the distal tendon shows scar changes and the central septum is retracted with “waving” pattern due to the retraction. (**B**)

Based on their location, rectus femoris injuries are classified as proximal injuries and middle third and distal injuries (Figs. 2, 3, 4, 5) [3, 19]. Rectus femoris has complex tendinous anatomy which should be addressed when deciding optimal treatment. The majority of these injuries are located in the central septum. [7, 20, 21] The central septum belongs to the proximal myotendinous junction of the rectus femoris. The proximal portion of this MTJ consists of two tendons: the direct or anterior tendon, and the indirect tendon. Ultimately, these tendons converge into a conjoined tendon. Distally, the connective tissue from the direct tendon is distributed along the anterior surface of the muscle continuing with the myofascial junction (epimysium, perimysium, and muscle fiber fascicles) [7, 20–22]. Its main function is at the beginning of the hip flexion [23]. The myoconective unit from the indirect tendon extends along the anterior midline of the muscle, forming the central septum. It later thins out and reach the lower third of the thigh, with a linear intramuscular shape. The indirect tendon performs its main function once the hip flexion has begun. [7, 20, 24]. The injury may be solely muscular, or it may primarily be associated with a tendon, such as the central tendon structure of the rectus femoris (Fig. 4) [4, 5]. This tendinous anatomy with aponeurosis structures runs throughout the length of rectus femoris [25]. Indirect muscle injuries are typically located close to interval between muscle and tendon tissue called a myotendinous junction (MTJ).

**Fig. 6:**
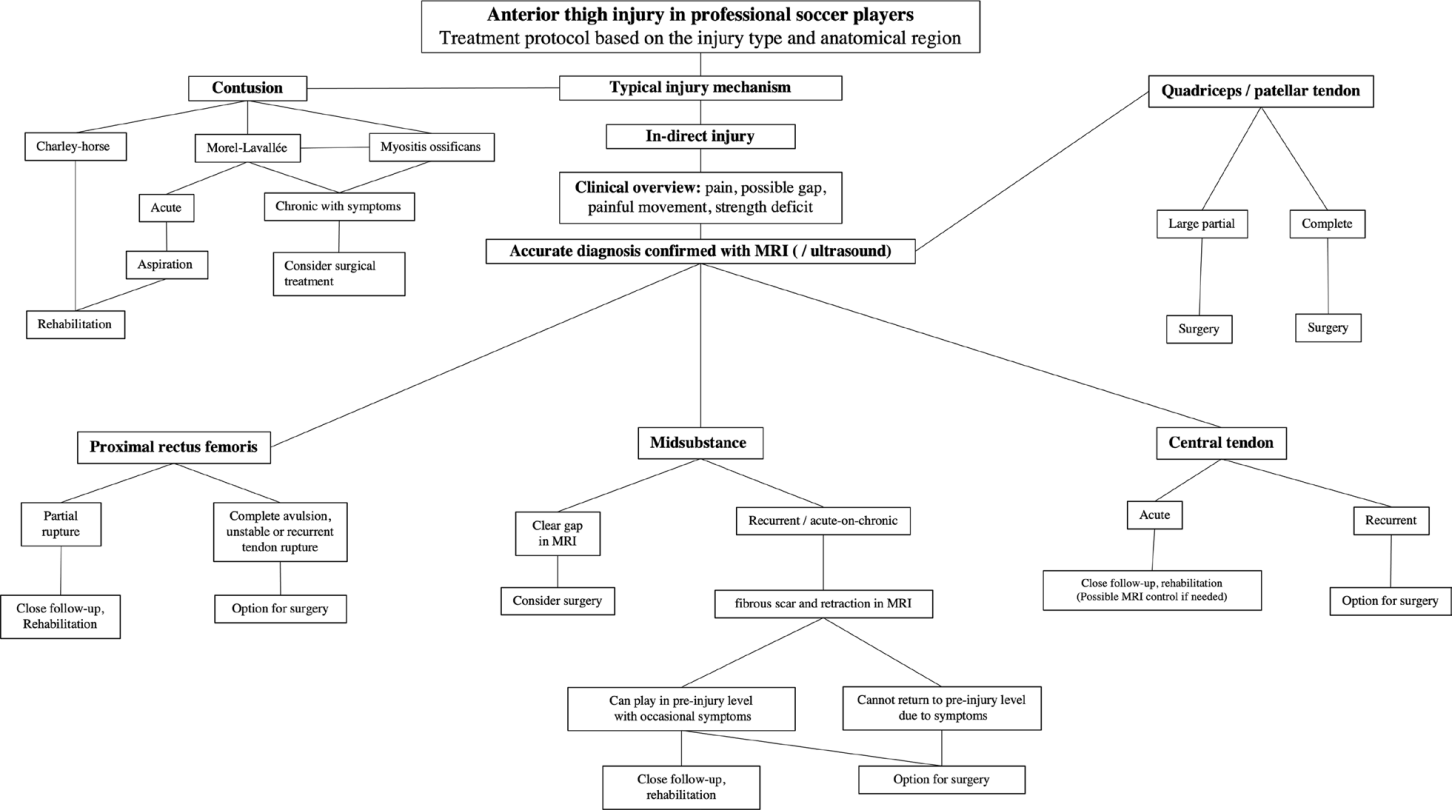
Treatment strategy of different muscle—tendon lesions of anterior thigh

Clinical work-up is summarized in the Fig. 6. The injured area is often sensitive to palpation, and if the rupture is considerable, it can also cause a visible and palpable gap on the skin. If the injury is located at the proximal part of the rectus femoris, the flexion of the hip against resistance is often painful and weaker compared with the non-injured side [26]. By contrast, injury in the middle and lower third of the muscle affects the extension strength of the knee. If the lower area of the quadriceps tendon near the patella is completely torn, it is often not possible to extend the knee against resistance. Even in cases of more extensive ruptures of the anterior thigh, a visible hematoma rarely develops [3]. On palpation, a chronic rupture of the central tendon of the rectus femoris muscle may feel the same as a muscle-related soft tissue tumor [27].

### Imaging

Imaging plays an important role in the evaluation and classification of anterior thigh injuries and in determining the course of treatment and assessing prognosis [28]. Magnetic resonance imaging (MRI) is the primary diagnostic method for early and correct diagnosis and to determine the grade of the injury [25]. To avoid the presence of blurring fibers due to the accumulation of blood, MRI should probably be performed as soon as possible after the injury, within 24–72 h and at least within 1–2 weeks postinjury when possible surgical intervention should be done [25]. Ultrasound (US) may also be used for anterior thigh injuries; if compared with MRI, the US is dynamic and offers a view of the injured area during muscle contraction and movement. US is also suitable for monitoring the healing process of injuries during rehabilitation [29]. By doing only US, it may be difficult to detect the small injuries located at the distal part of the MTJ, splits, or tendinous ruptures. Recent years, more investigation is done towards whether MRI imaging could predict return-to-play [30]. However, not clear consensus or recommendations can be given. If return-to-play imaging is conducted, MRI should be preferred modality [31]. The clinical picture with adequate strength balance and sport specific movements done pain-free are gold standard.

### Conservative treatment and rehabilitation

Most quadriceps muscle injuries heal conservatively (Fig. 6) [32–34]. The treatment follows the general principles of muscle injury treatment. The healing process for a muscle injury can be roughly divided into three phases: (1) degeneration of muscle fibers, the inflammation phase (1–3 days), (2) regeneration of muscle fibers (3–4 weeks), and (3) maturation and strengthening of muscle fibers, and the remodeling phase (3–6 months) [2, 35]. Initially PRICE (protection rest, ice, compression and elevation), and controlled loading of the affected muscle will help to minimize the extent of the injury and the size of the hematoma. After acute stage, pain-free active and passive stretching is started with isometric and eccentric contractions. These are followed with gym-based exercises. When gym-based treatments are passed, the rehabilitation progress is brought to field trainings. During field trainings, other gym exercises are continued, and loads are increased.

The key is to initiate rehabilitation early after the injury. To prevent muscle weakness and atrophy, subsequently, a controlled progression in loading and more demanding activities will be performed, avoiding pain to reduce the risk of reinjury [9]. It is important to avoid pain in injured muscles together with activation of the compensatory muscles as early as the initial stage and proceed progressively [10]. Rehabilitation should be implemented moderately and cautiously [9]. The return-to-play is always challenging and still without a full agreement [36], the greater agreement is about the complete resolution of the symptoms, full recovery of strength and performing capabilities evaluated by functional test and Global Positioning System, medical clearance, and even the biological time needed for the healing.

### Operative treatment

**Fig. 7:**
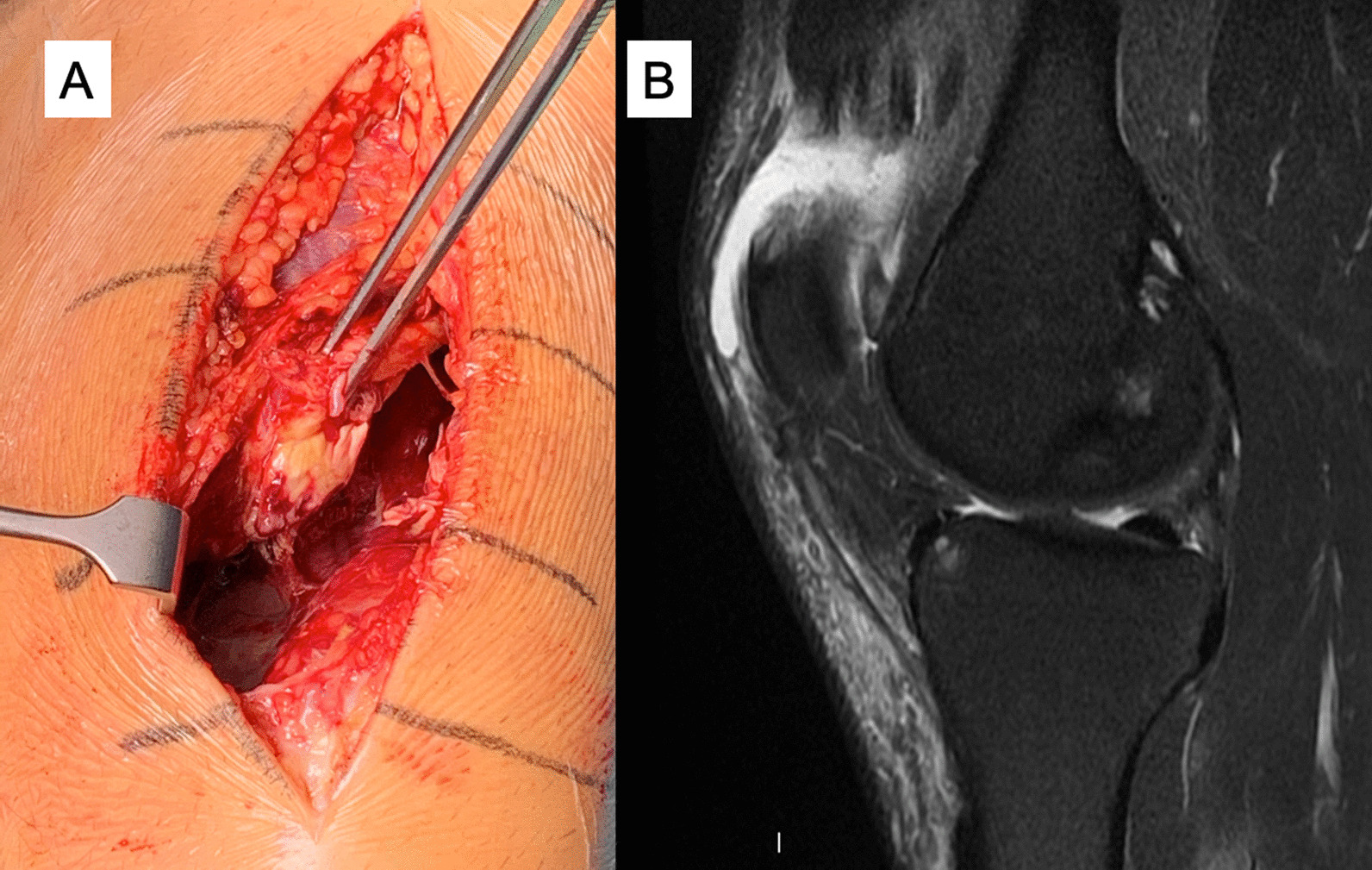
Quadriceps tendon complete rupture: operative image (**A**) and sagittal proton density fat saturated MRI image (**B**)

**Fig. 8:**
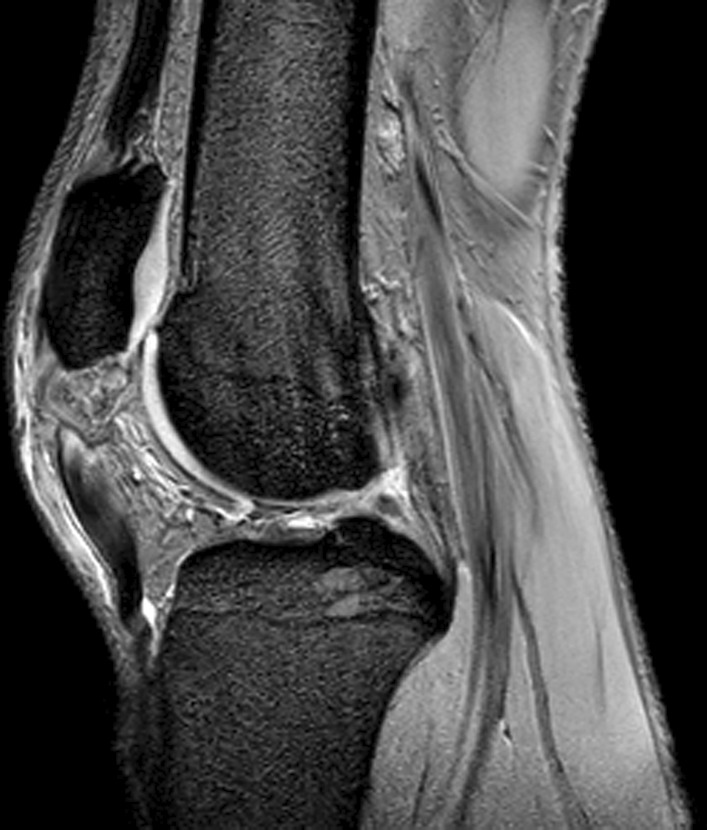
Sagittal gradient echo MRI image that shows patella-tendon complete proximal rupture. Proximal retraction of the patella

In some injuries the decision about treatment should be carefully evaluated, an initial well-managed operation with a planned and well supervised rehabilitation protocol will allow the athletes to return to the preinjury level of sports; avoiding a dramatic period of reinjuries affecting health and athletic performance when a conservative option is decided (Fig. 6). Operative treatment could be considered for a retracted (> 2 cm, curly tendon stump in MRI) proximal rectus femoris avulsion injury [3, 37–39], proximal recurrent tendon injury [40, 41], and in the complete rupture of the middle muscle belly area (Figs. 2, 3, 4) [13]. Rectus femoris central tendon rupture may sometimes also require operative treatment, especially in recurrent and chronic cases (Fig. 4) [5]. Without exception, total ruptures of the lower part of the quadriceps tendon and patellar tendon, as well as significant partial tendon ruptures, require operative treatment (Figs. 7, 8) [42–44]. Postoperative return-to-play takes 3–5 months depending on the extent of injury.

### Prevention

Improved mobility of the hip has been shown to reduce the risk of sustaining an anterior thigh injury, particularly in sports, including kicks [45]. If iliopsoas strength is reduced by 50% (measured in supine position), the rectus femoris starts to compensate for hip flexion, and therefore the rectus femoris could get overloaded and injured [7]. Also, preseason eccentric training could prevent anterior thigh injuries [46].

Exercises that improve the control of the core muscles of the body are also important in athletes' training, since good control of the pelvis reduces the burden on the tendon insertion of the rectus femoris, reducing the injury risk as well [7].

## Rare myotendinous injuries of the anterior thigh

Usually, when a patient presents pain in the anterior thigh, the physician will look for injuries in the rectus femoris, vastus medialis, and vastus lateralis muscles. However, sometimes the injury is localized in other myotendinous units, such as the tensor of the vastus intermedius or the sartorius muscle [47, 48]. These rare injuries can be diagnosed with US or MRI where blurring fibers, interstitial and intermuscular oedema can be visualized. These injuries can be treated non-operatively and the expected return-to-play is similar to other parts of anterior thigh injuries i.e. rectus femoris.

## Differential diagnoses and associated injuries

The anterior thigh injuries are not limited to muscle—tendon injuries only. Some entities are presented below.

### Myositis ossificans

Ectopic calcification, or myositis ossificans, may form inside the muscle due to contusion of the quadriceps muscle [49]. The cause of the formation of myositis ossificans is not known with certainty, but the risk of developing the condition is greater in large contusions than in smaller ones. Calcification is often clearly visible in the plain radiographs. It is important to minimize the size of the hematoma, quick restoration of the ROM, and prevention of recurrence of the injury. It is important not to initiate aggressive physiotherapy too soon, as it may increase the risk of developing myositis ossificans. A course of non-steroidal anti-inflammatory drugs may also reduce the risk of developing myositis ossificans [50–52]. The symptoms of myositis ossificans include dull pain and weakened muscle function. Myositis ossificans can increase the risk of re-injury by reducing muscle elasticity. Operative treatment often alleviates pain [52].

### Morel-Lavallée lesion

**Fig. 9:**
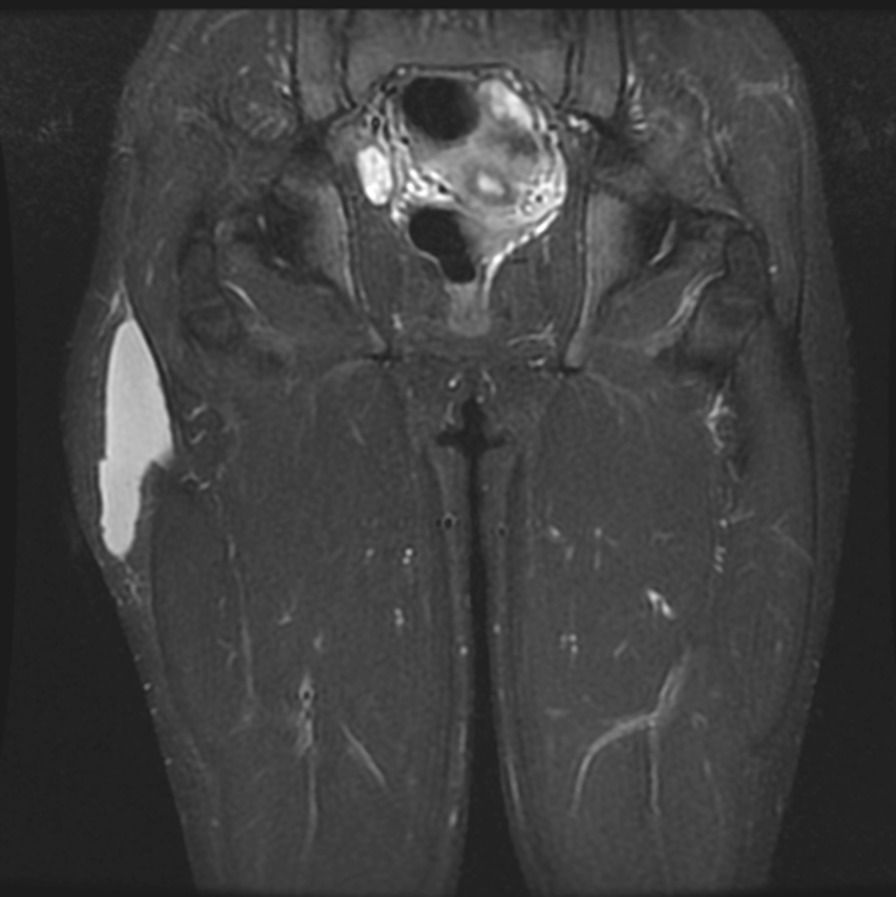
Coronal MRI STIR image that shows a chronic hematoma adjacent to the iliotibial tract, Morel-Lavallee lesion

In the Morel-Lavallée lesion, a contusion on the side of the thigh causes the subcutaneous fatty tissue to separate from the surface of the iliotibial tract. The lesion is often caused by a high-impact event (such as a fall), but it may also be sustained by soccer players, for example, because of kneeing or a sliding tackle. The difficulties in early diagnosis often cause a clinical problem, which worsens the prognosis of conservative treatment, resulting in the need for operative treatment later. MRI is the best method for diagnosing lesions (Fig. 9) [53].

## Typical pitfalls and future perspectives

Anterior thigh injury management can be challenging, and physician should be aware of common pitfalls. Incorrect decision making in early stages of injury is made, athlete could end up to series of acute—on—chronic injury pattern and loss a significant time of play. The most common pitfall in treating anterior thigh injuries is overlook them as simple muscle belly injuries and categorically misdiagnose these injuries as simple strains [4]. In recent years, research have shown that tendon related injuries possess high rate of prolonged rehabilitation, and structures such as central tendon has been discovered [5]. Physician treating these injuries are recommended to create their own regional pathway, where early diagnosis—using modern imaging such as MRI—is available together with musculoskeletal injury—oriented physiotherapist. As clinical picture and in-hand diagnosis could give suspicion of grading of injury, early follow-up visits are recommended. Also, patient history is relevant as multiple injuries—even small ones—might be due to lesions in tendon structures [7]. Flow chart shows practical management guide where different scenarios are shown (Fig. 6). More information is now available on surgical treatment and if a patient has a prolonged acute-on-chronic condition with multiple sequences, surgery may be possible and should not be ruled out. Distal quadriceps, patella tendon injuries and retracted proximal rectus femoris injuries are typically treated in acute phase surgery, whereas partial tears affecting other parts of anterior thigh injuries could be managed with first line non-operative means.

The future perspectives will focus on injury prevention as well as rehabilitation of different injury type. More clinical tools such as video analysis during game play are under investigation [54]. Rising evidence and knowledge is coming from imaging diagnostics, in order to more precisely predict return-to-play time.

## Summary

Anterior thigh injuries are common in sports, especially soccer. Although most of these injuries respond well to conservative treatment, clinicians should be aware when treating these injuries. Tendon injuries may be underestimated, which will considerably compromise rehabilitation. The injury may also recur easily if the initial diagnosis and treatment method are incorrect. The clinician should also keep in mind other causes of pain in the anterior thigh area, since not all pain is caused by muscle or tendon injuries.

## Data Availability

The datasets during and/or analysed during the current study available from the corresponding author on reasonable request.

## Abbreviations

MRI: Magnetic resonance imaging
US: Ultrasound
ROM: Range of motion
